# Control of carbapenem-resistant *Acinetobacter baumannii* outbreak in an intensive care unit of a teaching hospital in Southern Italy

**DOI:** 10.1186/s12879-016-2036-7

**Published:** 2016-12-12

**Authors:** Aida Bianco, Angela Quirino, Mariavalentina Giordano, Vito Marano, Claudia Rizzo, Maria Carla Liberto, Alfredo Focà, Maria Pavia

**Affiliations:** Department of Health Sciences, University of Catanzaro “Magna Græcia”, 88100 Catanzaro, Italy

**Keywords:** *Acinetobacter baumannii*, Extensively drug resistance, Intensive care unit, Infection control, Outbreak, Rep-PCR

## Abstract

**Background:**

*Acinetobacter baumannii* is an opportunistic pathogen that has become a major cause of concern, since it is a frequent cause of healthcare-associated infections (HAIs). The aim of the study was to describe the occurrence, the management and the control of an outbreak that occurred in an intensive care unit (ICU) of a teaching hospital in Southern Italy caused by multiple strains of extensively drug-resistant *A. baumannii* (XDRAB).

**Methods:**

Case-patient was defined as a patient with an healthcare-associated infection caused by an XDRAB isolate identified in a clinically significant culture. Environmental samples were collected from different surfaces. The isolates were identified by typical Gram stain morphology, using the Vitek 2 system (bioMérieux, France) and by MALDI-TOF MS mass spectrometry (bioMèrieux, France). Genotyping was performed through rep-PCR analysis.

**Results:**

A patient presented an XDRAB ventilator-associated pneumonia at admission and was managed with strict isolation precautions until discharge. Five patients had a ventilator-associated pneumonia and two had a central line-associated bloodstream infection. Of the environmental samples, 1 sample obtained from the side of the bed of an infected patient yielded growth of XDRAB. Infection control measures were adopted. Rep-PCR analysis identified four patterns.

**Conclusions:**

The integration of epidemiological and microbiological data and the application of infection control measures were crucial to bring such an outbreak to a rapid halt. The distinctive characteristic of this study was the complex molecular pattern of the outbreak, which subsided in a short period of time due to adherence to infection-control measures, confirming the fundamental role of molecular typing in the comprehension of outbreaks dynamics and of integrated control interventions for the interruption of epidemic events.

**Electronic supplementary material:**

The online version of this article (doi:10.1186/s12879-016-2036-7) contains supplementary material, which is available to authorized users.

## Background

*Acinetobacter baumannii* is an opportunistic pathogen that has become a major cause of concern, since it is a frequent cause of healthcare-associated infections (HAIs), particularly lower respiratory tract, bloodstream, and urinary tract infections [1]. This micro-organism is responsible for up to 10% of all Gram-negative bacterial infections in intensive care units (ICUs) in Europe and the USA [2, 3]. *A. baumannii* HAIs have been associated with mortality rates ranging from 5% in general hospital wards to 54% in ICUs [4].

The organism’s ability to survive under a wide range of environmental conditions and to persist for extended periods of time on surfaces makes it a frequent cause of outbreaks and an endemic, healthcare-associated pathogen [5]. Outbreaks caused by extensively drug-resistant *A. baumannii* (XDRAB) have been identified in several ICUs worldwide (up to 30% of clinical isolates) and in many instances such isolates are resistant to the vast majority of available antimicrobial agents, including carbapenems [6]. As reported in Maragakis et al. [7], XDRAB is defined as organisms that show non-susceptibility to at least one agent in all but two or fewer antimicrobial categories [7].

Outbreaks of XDRAB have been described in ICUs of hospitals in several countries [8–11]. XDRAB outbreaks in various wards have also been described in Italian hospitals [11, 12].

Herein, we describe the occurrence, the management and the control of an outbreak of infection caused by multiple strains of XDRAB, which occurred in an ICU of a teaching hospital located in Southern Italy.

## Methods

### Clinical setting

The outbreak involved eight patients admitted to the ICU of a teaching hospital located in Calabria (Italy) between March and May 2014. The hospital has 165 beds and hosts several wards, including medical and surgical specialties. The ICU has 8 beds, with two single rooms for isolation. Since May 2013, in the ICU, an active surveillance of HAIs has been continuously carried out by trained personnel of the Hospital Hygiene Unit. Moreover, based on a multi-drug resistant bacteria infections prevention protocol, patients are screened for three selected multi-drug resistant (MDR) bacteria with nasal swabs for detection of methicillin - resistant *Staphylococcus aureus*, and rectal in combination with oro-pharyngeal swabs for detection of MDR *A. baumannii* and MDR *Klebsiella spp.* at admission and then on a weekly basis.

### Identification and investigation of the outbreak

The outbreak was immediately detected throughout the active patient-based surveillance of HAIs. Demographic and clinical data were gathered for all ICU patients, including: age, gender, diagnosis upon ICU admission, admission type, underlying disease, transfer from other wards or hospitals, duration of ICU stay, invasive procedures (surgical procedures, central venous catheter, etc.), antimicrobial therapies (choice of antibiotic, dosage, regimen, route and time of drug administration), presence of colonization or infection, date and site of XDRAB isolation and outcome. Case-patient was defined as a patient with an HAI caused by an XDRAB isolate identified in a clinically significant culture. HAI was defined according to the criteria used by the Centers for Disease Control and Prevention [13–15]. Clinical episodes of colonization or infection were considered hospital acquired if they were not present at the time of admission and appeared 48 h after hospital admission. Colonization with XDRAB was defined as the detection at admission or the acquisition of this pathogen during hospitalization, but without any evidence of clinical disease.

The investigation was considered to be an infection control response, and as such, it was not subjected to approval by an institutional review board or required written informed consent from patients.

### Environmental investigation

To identify the extent of environmental contamination and, eventually, any possible source of the outbreak, environmental samples were collected using Nutrient agar or Mac Conkey (Liofilchem srl—Roseto degli Abruzzi TE, Italy) agar plates. The samples were collected from floors, medical facilities, furnishings, mechanical ventilator equipment, monitors, surfaces of tables for carrying medical instruments, patients’ charts, and hands of randomly selected ICU’s HCWs. The ambient air was also examined, using the Microflow α microbiological air sampler (Aquaria srl, Lacchiarella—Milan, Italy).

### Outbreak management

We intensified several infection control measures, such as isolation of colonized or infected patients with XDRAB and contact precautions and terminal disinfection procedures were carefully performed. Then, environmental cleaning and disinfection as well as the sterilization process of reusable equipment were reviewed. Furthermore, during the outbreak, meetings with all HCWs were held to discuss about any potential flaws in standard ICU practices and to provide information and training periods.

### Description of the outbreak chronology

We reconstructed the chronological distribution of the infection cases considering several factors including hospitalization of patients in chronologically compatible periods, length of stay, administered therapies or performed diagnostic investigations, medical consultations and procedures by healthcare personnel from other departments, such as dialysis unit.

### Microbiologic methods and molecular typing

*Acinetobacter* isolates were conventionally identified by typical Gram stain morphology, using the Vitek 2 system (bioMérieux, France) to evaluate antibiotic susceptibility and by MALDI-TOF MS mass spectrometry (bioMèrieux, France) [16]. The results were interpreted according to the Clinical and Laboratory Standards Institute (CLSI) criteria [17]. Resistance to carbapenems was also performed by the E-test method.

Genotyping was performed by rep-PCR analysis using as references an environmental *A. baumannii* strain previously isolated from Mediterranean sea water sample and ATCC 19606. Isolated *A. baumannii* were grown on Columbia blood agar; DNA was extracted from a 10-μl loopful of each bacterial colony, using an UltraClean Microbial DNA isolation kit (Mo Bio Laboratories, Carlsbad, CA). The extracted DNA was amplified using a DiversiLab *Acinetobacter* DNA fingerprinting kit (bioMèrieux, France), following the manufacturer's instructions. The method uses two interpretation criteria: Pearson correlation, based on the intensity of electrophoretic bands, and Kulbach correlation, focused on the height and width of the peaks [18].

## Results

### Demographic and clinical characteristics of infected patients

Infected patients’ characteristics are presented in Table 1. Positivity to XDRAB appeared on average 8.3 days ± 8.8 (range 0-28 days) after hospitalization. All patients had undergone mechanical ventilation, tracheostomy, central venous catheter and/or indwelling urinary catheter. At the time of first XDRAB isolation, the mean duration of mechanical ventilation was 7.8 ± 8.2 days. Antibiotics had been prescribed for treatment or prophylaxis in all patients but two (Patients 1 and 3) before XDRAB isolation. One patient was already infected and another was colonized by XDRAB at admission to ICU.

**Table 1:** Demographic and clinical characteristics of patients with positive isolates for *XDRAB*

Variable	No of patients (%) *n* = 8
Age (years), mean ± SD	64.5 ± 14.7
Male %	4 (50)
Intrinsic Risk Factors	
Tabagism	3 (37.5)
Diabetes Mellitus	2 (25)
Chronic Obstructive Pulmonary Disease	2 (25)
Previous hospitalization	2 (25)
Chronic kidney disease	1 (12.5)
Malignancy	1 (12.5)
Politrauma	1 (12.5)
Use of steroid	7 (87.5)
Diagnoses upon admission	
Coronary Artery Disease	2 (25)
Respiratory Failure	1 (12.5)
Pulmonary Embolism	1 (12.5)
Pneumonia	1 (12.5)
Cancer	1 (12.5)
Shock	1 (12.5)
Fracture/Politrauma	1 (12.5)
Site of isolates	
Bronchoalveolar lavage	6 (37.5)
Endotracheal aspirate	4 (25)
Central venous blood	2 (12.5)
Sputum culture	2 (12.5)
Peripheral venous blood	1 (6.25)
Rectal swab	1 (6.25)
Device	
Endotracheal tube	8 (100)
Central venous catheter	8 (100)
Pleural dreinage	2 (25)
Total Parental Nutrition	5 (62.5)
Previous use of antibiotic in the preceding 90 days	
β-lactam	1 (12.5)
β-lactam plus another antibiotic	1 (12.5)
β-lactam plus at least two other categories of antibiotics	6 (75)
Hospital stay before acquisition of XDRAB (days)	8.3 ± 8.8
ICU stay before acquisition of XDRAB (days)	9.4 ± 8.8
Clinical significance	
Ventilator associated Pneumonia	6 (75)
Central venous catheter infection	2 (25)
Outcome	
In-hospital mortality	4 (50)

### Outbreak description

**Fig. 1:**
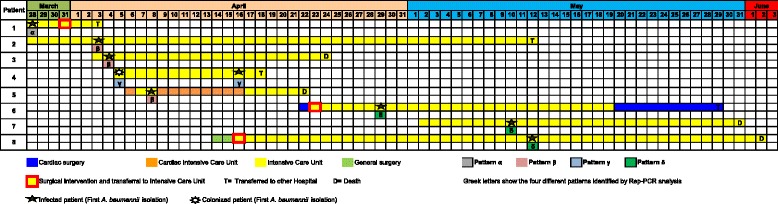
Chronogram. Distribution of *XDRAB* positive patients as a function of time and ward

As it is shown in Fig. 1, between March (first case on March 28, 2014) and May 2014 (last case on May 12, 2014), eight patients hospitalized in the ICU developed XDRAB infections. *A. baumannii* clinical isolates*,* identified by the Vitek 2 system, were confirmed by m/z 5747/5749 range peaks analysis using MALDI-TOF MS mass spectrometry (data not shown).

The first isolated patient was a 57-year-old woman, transferred on March 28^th^ to our hospital from a tertiary hospital located in Calabria region. The patient was affected by a ventilator-associated pneumonia at admission caused by an XDRAB. She was managed with strict isolation precautions until discharge. During the following 2 months, other seven patients developed XDRAB infections. Specifically, five patients (Patients 2, 3, 5, 6 and 7) had a ventilator-associated pneumonia and two (Patients 4 and 8) a central line-associated bloodstream infection. Patient 4 was colonized with XDRAB isolated from a rectal swab upon ICU admission and, afterwards, XDRAB was isolated from this patient’s blood.

All the isolates were resistant to aminoglycosides, trimethoprim-sulphamethoxazole, antipseudomonal carbapenems, antipseudomonal fluoroquinolones, antipseudomonal penicillins plus β-lactamase inhibitors, extended-spectrum cephalosporins, folate pathway inhibitors, penicillins plus β-lactamase inhibitors and tetracyclines, whereas they were susceptible to colistin, with a minimum inhibitory concentration of 0.5 mg/mL. All isolates were susceptible to ampicillin-sulbactam (MIC ≤ 4 mg/L) except for the isolates from Patient 4 (ACI 3414 and ACI 4314) showing an MIC equal to 12 mg/L.

All strains were resistant to carbapenems (ertapenem, imipenem, meropenem). In particular, for all isolates, minimum inhibitory concentrations to carbapenems ranged between 8 and 16 μg/mL, confirming they were resistant to this category of antibiotics, given that the breakpoint is ≥ 8 μg/mL [17]. Resistance to carbapenems was confirmed by the E-test for all strains except for that isolated from Patient 1 (ACI 3014) that was susceptible to meropenem and imipenem.

Four patients died due to multiple concomitant medical problems and four patients were transferred to other wards/hospitals.

Only one environmental sample obtained from the side of the bed of an infected patient (Patient 8) yielded growth of XDRAB.

### Adopted infection control measures

Several infection control measures were promptly adopted, including:

- single room isolation of patients or cohorting of colonized/infected patients in designated areas of the ICU, allocation of dedicated materials for patient care;
- enhancement of contact precautions to interrupt transmission, including alcohol based hand rub and use of disposable gloves and gowns;
- periodic meetings with all nursing and ancillary staff to provide information and training regarding the identified critical areas and operational and technical procedures;
- compulsory placement of medical devices and personal protection devices (overalls, gloves, etc.) at the point where the patient is assisted for immediate access, in both structural and functional isolation;
- strict respect of behavioral protocols, such as hand hygiene, clothing, cleaning and terminal disinfection of the environment and surfaces, disinfection and sterilization of reusable medical devices by healthcare workers from inside and outside the ICU;
- evaluation of the process of environmental sanitation and adoption of specific disinfection procedures using, whenever possible, sodium dichloroisocyanurate at 1000 ppm available chlorine or, alternatively, 70% alcohol, as indicated in the 2013 Guidelines of the Health Protection Agency [19].

### Molecular typing of *A. baumannii* isolates

**Fig. 2:**
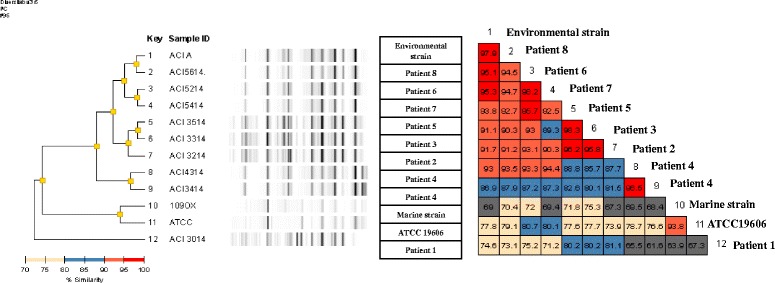
Dendrogram plot. Results of molecular typing of *A. baumannii* isolates and percentage of similarity

Rep-PCR analysis identified four patterns (pattern α, β, γ, δ), as shown in Fig. 1 and in the dendrogram (Fig. 2).

*A. baumannii* isolated from Patient 1 was the only strain classified in pattern α, as well as the strains isolated from Patient 4 (one from a rectal swab at admission and the other from blood) that were the only isolates classified in pattern γ. Bacterial strains from Patients 2, 3 and 5, isolated from 3 to 8 of April, were similar or indistinguishable when their genome fingerprints were compared (pattern β). Similarly, strains from Patients 6,7 and 8, isolated from April 29 to May 12 were similar or indistinguishable and were grouped in pattern δ. Moreover, *A. baumannii* isolated from Patient 8 was indistinguishable (similarity of 97.9%) when compared with the environmental strain isolated from her bed and isolates from Patients 6 and 7 were similar (similarity > 95%) to the same environmental strain. Finally, the strains isolated from Patient 5 and Patient 6 were similar (similarity = 95.7%) although they were classified in patterns (β and δ).

## Discussion

This study reports the spread and containment of different XDRAB strains that caused an outbreak in an ICU in Italy. The integration of epidemiological and microbiological data and the strict application of infection control measures have played a decisive role to interrupt the spread of XDRAB among patients.

The prompt application of the organizational measures, such as isolation of colonized/infected patients avoided the spread of the bacterial strains to other patients and reduced the dissemination in the environment. In addition, multiple rigorous cleaning and disinfection interventions contributed to the successful eradication of the microorganism from the environment. Finally, we highlighted the importance of standard and contact precautions, with particular attention given to hand-washing with alcohol-based solutions during the management of patients and change of gloves, gowns and other personal protective equipment used during the care of colonized/infected patients.

Of note is our finding that the strains involved in this outbreak were resistant to at least one agent in the carbapenems category, that were considered to be the only antibiotics with reliable activity against *A. baumannii* [20], and they were fully susceptible to colistin. The increasing use of colistin among carbapenems resistant *A. baumannii* infected patients is a cause of concern because it may lead to the emergence of resistance. In view of the emergence of colistin resistance, antibiotic stewardship was also advocated during this outbreak investigation as an additional intervention to reduce further selection of resistance in circulating *A. baumannii* strains.

Clinical and environmental isolates showed a very similar pattern of antimicrobial susceptibility, suggesting that the first patient, infected upon admission to the ICU, was the index case and the source spreading the strain to other patients and to the environment. However, molecular typing showed a substantially more complex scenario that, integrated with the epidemiological, clinical and chronological data, provided useful evidence to comprehend the evolution of the outbreak. With respect to the identified pattern, it should be noted that all strains but one corresponding to the first infected patient (Patient 1) showed a DNA fingerprinting similarity above 85%, therefore, they could be considered genetically related. We may hypothesize a genetic evolution of the isolates.

The strengths of the study lie in the small number of patients involved in the outbreak, probably attributable to the infection control programme in place in the centre, in the long investigation period that allowed to evaluate the effects of the control measures and in the regular staff training programs that were task oriented and continuously reinforced.

This investigation has several limitations that should be noted. First, the study was not able to evaluate the information regarding administration of carbapenems before XDRAB detection that, as suggested by the findings of previous studies [21, 22], could be one of the risk factors for XDRAB acquisition. Second, we did not apply sequencing techniques. Instead, we used the DiversiLab system, that has demonstrated to be an accurate and reliable typing method particularly vocated to outbreak investigations [23–26]. Third, this study was carried out in a descriptive manner, since we could not perform a case–control study due to the small number of colonized/infected patients, so associations between exposure to certain risk factors and XDRAB acquisition could not be detected.

## Conclusions

In conclusion, the distinctive characteristic of the present study was the complex molecular pattern of the outbreak, which subsided in a short period of time due to strict adherence to infection control measures, confirming the fundamental role of molecular typing in the comprehension of outbreak dynamics and of integrated control interventions for the interruption of epidemics.

## Additional file

Additional file 1: Dataset. (XLSX 18 kb)

## Abbreviations

HAI: Healthcare-associated infection
HCW: Healthcare worker
ICU: Intensive care unit
MDR: Multi-drug resistant
XDRAB: Extensively-drug resistant *Acinetobacter baumannii*
